# Toxicity of a plant based mosquito repellent/killer

**DOI:** 10.2478/v10102-012-0031-4

**Published:** 2012-12

**Authors:** Bhoopendra Singh, Prakash Raj Singh, Manoj Kumar Mohanty

**Affiliations:** 1 Dept. of Forensic Medicine & Toxicology, Rajendra Institute of Medical Sciences, Ranchi, India; 2 Dept of Physiology, Sampurnanand Sanskrit University, Varanasi, India; 3 Dept. of Forensic Medicine & Toxicology, Sri Venkateshwaraa Medical College and Research Centre Ariyur, Pondicherry, India

**Keywords:** mosquito repellent, herbal, mosquito out, toxicity

## Abstract

The mission to make humans less attractive to mosquitoes has fuelled decades of scientific research on mosquito behaviour and control. The search for the perfect topical insect repellent/killer continues. This analysis was conducted to review and explore the scientific information on toxicity produced by the ingredients/contents of a herbal product. In this process of systemic review the following methodology was applied. By doing a MEDLINE search with key words of selected plants, plant based insect repellents/killers pertinent articles published in journals and authentic books were reviewed. The World Wide Web and the Extension Toxicity Network database (IPCS-ITOX) were also searched for toxicology data and other pertinent information. Repellents do not all share a single mode of action and surprisingly little is known about how repellents act on their target insects. Moreover, different mosquito species may react differently to the same repellent. After analysis of available data and information on the ingredient, of the product in relation to medicinal uses, acute and chronic toxicity of the selected medicinal plants, it can be concluded that the ingredients included in the herbal product can be used as active agents against mosquitoes. If the product which contains the powder of the above said plants is applied with care and safety, it is suitable fo use as a mosquito repellent/killer.

## Introduction

The quest to make humans less attractive to mosquitoes has fuelled decades of scientific research on mosquito behaviour and control worldwide. Yet mosquitoes will transmit disease to more than 700 000 000 people annually and will be responsible for the deaths of 1 of every 17 people currently alive (Taubes, 1977). Malaria results from infection with a protozoan carried by mosquitoes and according to reports from the World Health Organization (WHO), it causes as many as 3 000 000 deaths annually (Shell, 1997). In India, more than 1.75 million malaria cases were reported due to infection with a protozoan carried by mosquitoes, causing more than 1 000 deaths annually (Park, 2005). Mosquitoes transmit the arboviruses responsible for yellow fever, dengue haemorrhagic fever, epidemic polyarthritis, and several forms of encephalitis (some of which are found in India).

Historically, the strategies for reducing the incidence of mosquito-borne disease have been two-pronged, centring on habitat control (through chemical and biological means) and the use of personal protection in the form of insect repellents/killers. This article reviews the scientific data on natural (plant-derived) insect repellents/killers currently available and the related toxicity.

Mosquitoes are found all over the world, except in Antarctica. These two-winged insects belong to the order Diptera. Members of the genera *Anopheles*, *Culex*, and *Aedes* are most commonly responsible for bites in humans. To develop, mosquitoes require an environment of standing water. As a group, they have adapted to complete their life cycle in diverse aquatic habitats, including fresh water, salt-water marshes, brackish water, water found in containers, old tires, or tree holes, etc. The life cycle of the mosquito has four stages. The female mosquito lays her eggs, up to several hundred at a time, on the surface of the water or in an area subject to flooding. Unhatched eggs of some species can withstand weeks to months of desiccation, remaining viable until the right conditions for hatching occur. The eggs of most species hatch in 2 to 3 days, and the larvae feed on organic matter in the water for about a week until they change into pupae. The pupae live at the surface of the water for 2 to 3 days before metamorphosing into adult mosquitoes.

Only female mosquitoes bite. Male mosquitoes feed primarily on flower nectar, whereas female mosquitoes require a blood meal to produce eggs. They usually feed every 3 to 4 days; in a single feeding, a female mosquito typically consumes more than its own weight in blood (Clements, 1963). Certain species of mosquitoes prefer to feed at twilight or night time; others bite mostly during the day.

Despite the obvious desirability of finding an effective oral mosquito repellent, no such agent has been identified (Khan *et al.*, 1969; Strauss *et al.*, 1968). Thus, the search for the perfect topical insect repellent/killer is continued. Efforts to find such a compound have been hampered by the numerous variables that affect the inherent repellency of any chemical. The repellents do not act with a single mode of action, furthermore little is known about how repellents act on their target insects (Davis, 1985; Wright, 1975). Moreover, different species of mosquitoes may react differently to the same repellent (Rutledge *et al.*, 1983).

Many factors play a role in how effective any repellent is, including the frequency, dosage and uniformity of application, the number and species of the organisms attempting to bite, the user's inherent attractiveness to blood-sucking arthropods, and the overall activity level of the potential host (Schreck, 1995).

To find out the suitable mosquito repellent/killer thousands of plants have been tested as sources of insect repellents/killers. Even though none of the plant-derived chemicals tested to date demonstrates the broad effectiveness and duration of protection of DEET, a few of them do show repellent activity (Sukumar *et al.*, 1991; Trongtokit *et al.*, 2005). The plants whose essential oils have been reported to have repellent activity include citronella, neem, cedar, verbena, pennyroyal, geranium, lavender, pine, cajuput, catnip, cinnamon, rosemary, basil, thyme, allspice, garlic, and peppermint. Unlike synthetic insect repellents, plant-derived insect repellents have been relatively poorly studied. When tested, most of the essential oils yield short-lasting protection, lasting from a few minutes to as long as 2 hours.

A herbal composition prepared by a local manufacturer mentioned in the section of Materials and Methods would repel/kill multiple species of biting arthropods and remain effective for a period of 4 months (as claimed by the manufacturer), without causing any severe toxicity. The aim of writing this article was to explore the scientific information pertaining to toxicity produced by the ingredients/contents of the product.

## Materials and methods

The information on the subject was explored by using MEDLINE search with key words of selected plants and plant based insect repellents/killers, concerning pertinent articles published in English language journals. Furthermore, the World Wide Web and the Extension Toxicity Network database (IPCS-ITOX), different books and journals available in the library of the medical institution were also searched for toxicology data and other pertinent information.

One packet of the herbal based product “Mosquito Out” contains the following plant/herb powders as active ingredients (Table 1).

**Table 1:** Components of Mosquito Out.

No.	Local name	Scientific Name	Quantity (in grams)
1	Neem powder	*Azadirachta indica*	2.0
2	Salvia	*Salvia fruticosa*	2.0
3	Akkalkara	*Pyrethrum radix*	1.0
4	Dhoradvan (Shankhapuspi)	*Convolvulus pluricaulis*	1.0
5	Karanj	*Pongamia pinata*	0.5
6	Sabja (Tulsi)	*Ocimum basilicum*	0.5
7	Nirgudi	*Vitex negundo*	0.5
8	Sal (Sakhu)	*Shorea robusta*	0.5
9	Karamkas (Kamarkas)	*Salvia plebeian*	1.0
10	Soap stone powder	Hydrated magnesium silicate	1.0

### Description and Toxicity of Components

Insecticides derived from plants are believed to be relatively safe and biodegradable, and they are readily available sources of (Shell, 1997). There is however no place for belief in science and the data have to be experimentally proved and scientifically analysed. To detect the harmful effects of the components/ingredients of the above mentioned product, the toxicological properties have been reviewed and are presented in this article.

## Results and discussion

### Neem (Azadirachta indica) Powder

The Neem tree (Scientific Name – *Azadirachta indica*) provides many useful compounds that are used as pesticides and can be used as mosquito repellent/killer. Since ancient times, Neem has been associated with healing in the subcontinent of India. Its ingredients have been reported to have several beneficial health effects, such as blood sugar lowering properties, anti-parasitic, anti-inflammatory, anti-ulcer and hepato-protective effects. A large number of medicinals, cosmetics, toiletries, and pharmaceuticals are now based on Neem derivatives due to its unique properties. The main active ingredient of Neem is Azadirachtin. Azadirachtin has been shown to inhibit larval, pupal and adult moults and reproduction of both plant feeding and aquatic larvae like mosquitoes. It does not act on the digestive or nervous system and does not lead to development of resistance in future generations (Rutledge *et al.*, 1983). One-part per million (1 ppm) concentration of Azadirachtin is sufficient to produce almost 100% larval mortality **(**Schreck *et al.*, 1995).

#### Acute Toxicity

Table 2. shows the lethal dose for the active ingredient of Neem tree (Azadirachtin).

**Table 2:** The lethal dose for the active ingredient of Neem tree (Azadirachtin).

Animal	Route of Exposure	LD_50_
Rat	Oral	>2 000 mg/kg bw
Rat	Dermal	>2 000 mg/kg bw
Rat	Inhalation	>2.11 mg/L
Rabbit,	Ocular	Mild irritant to conjunctiva
Guinea Pig	Dermal Sensitisation	Not a sensitiser

#### Chronic toxicity

Raizada *et al.* (2001) assessed the subchronic toxicity of Azadirachtin in rats. Oral ingestion of Azadirachtin at doses of 500, 1000, and 1500 mg/kg/day for 90 days to male and female rats did not produce any signs of toxicity, mortality, changes in tissue weight, pathology, serum and blood parameters. Therefore the highest dose 1500mg/kg/day of Azadirachtin could be used as a basal dose for the determination of the oral No-Observed-Adverse-Effect Level (NOAEL) for 90 days to calculate its safety margin. The topical exposure to sublethal doses of Azadirachtin did not result in any significant alterations in acetylcholinesterase (AchE) activity in the nervous system of the cockroach (Shafeek *et al.*, 2004).

Ther estimated safe dose (ESD) for non-aqueous extracts of the Neem based product is 0.002 to 12.5µg/kg bw/day, and for unprocessed materials seed oil or the aqueous extracts the ESD is 0.26 to 0.3mg/kg bw/day, 2µl/kg bw/day respectively). For the pure compound Azadirachtin the ESD is 15 mg/kg/day. The use of Neem derived pesticide as a mosquito repellent should not be discouraged (Boeke *et al.*, 2004).

### Salvia (Scientific Name – *Salvia fruticosa*)

There are many species of Salvia grows and available in India. Powder of the Salvia fruticosa plant is another ingredient of the product studied. Salvia fruticosa has shown antiimplantation, antifertility and reproductive toxicity potentials after ingestion of aqueous and ethanolic extracts of leaves in male and female rats (Sonboli *et al.*, 2006). The extract of S. lavandulaefolia Vahl has shown anticholinesterase, antioxidant, anti-inflammatory, oestrogenic and CNS depressant (sedative) effects, all of which are currently relevant to the treatment of Alzheimer's disease (AD) (Qureshi *et al.*, 1989). The aqueous extract of the root of Salvia heamatodes possesses significant cardiotonic and anticonvulsant activities. The aqueous extract of Salvia leriifolia seed has shown anti-nociceptive and anti-inflammatory activity in rats (Shafeek *et al.*, 2004). The extract of an another species (*S. transsylvanica)* induced significant analgesic, antipyretic, antiepileptic, antiinflammatory, antiulcerogenic, as well as tranquillizing activities, besides increasing the bleeding time and exhibiting no central skeletal muscle relaxant effect compared with control groups and standard drugs. The oil of three Salvia species, i.e. S. santolinifolia, S. hydrangea and S. mirzayanii showed antimicrobial activity (Akber *et al.*, 1985).

#### Acute toxicity

The lethal dose (LD50) is different for active the ingredient of different Saliva species extracts. The LD50 of the extract of S. fruticosa plant is 4.437 g/kg b.w. (Perry *et al.*, 2003). The LD50 of the extract of Salvia leriifolia was found to be 19.5 g/kg b.w. (i.p.) in mice (Hosseinzadeh *et al.*, 2003) and that of S. splendens is 1287.3 mg/kg b.w. (Qureshi *et al.* 1989).

#### Chronic toxicity

Toxicological studies carried out on aqueous extract of Salvia splendens commonly known as Red Sage revealed that the active ingredient was toxic only in higher doses and caused hemorrhages. The aqueous root extract of Salvia splendens increased the clotting time of dog plasma by increasing the normal prothrombin time from 10–15seconds to 35 seconds. Salvia splendens was found to possess anticoagulant property (Qureshi *et al.*, 1989).

The extract of Salvia officinalis showed a certain degree of larval toxicity by significantly affecting the growth indexes (relative growth rate (RGR), efficiency of conversion of ingested food (ECI), and efficiency of conversion of digested food (ECD) (Maklad *et al.*, 1999).

### Pyrethrum radix (Akkalkara)

Pyrethrum radix is locally known as Akkalkara. It contains Pyrethrin, which is an active chemical of the Pyrethrum plant. The main active constituents of Pyrethrum are pyrethrin I and pyrethrin II, with smaller amounts of the related cinerins and jasmolins.

#### Acute toxicity

“Pyrethrum” extracts have not been classified by the WHO for acute toxicity. Table 3 shows the lethal dose for the active ingredient of Pyrethrum plant (Pyrethrin).

**Table 3:** The lethal dose for the active ingredient of Pyrethrum plant (Pyrethrin).

Animal	Route of Exposure	LD_50_
Rat	Oral	>1 200 mg/kg bw
Rabbits	Dermal	>2 000 mg/kg bw
Rat	Inhalation	>3.4 mg/L
Rabbit	Ocular Irritation	Minimally
Rabbit	Dermal Irritation	Minimally
Guinea Pig	Dermal Sensitisation	Not a sensitiser

#### Toxicity – human data

Pyrethrins have a wide margin of safety when used judiciously, and there are few adequately documented cases of fatal pyrethrin poisoning in man. It appears that the main toxicity to humans is related more to the solvent vehicle (Gosselin et al 1984). Injury is more likely to result from allergy than direct toxicity. The amount immediately dangerous to life or health: 5000 mg/cubic metre (NIOSH 1997). The minimal lethal dose of pyrethrins is probably in the region of 10 to 100 grams. Animal studies suggest that the young are at greater risk than adults.

#### Chronic toxicity

A 13-week study for toxicity of pyrethrum plant extract in mice, rats, and dogs showed statistically significant decreases in mean body weight or body-weight gain at high doses throughout most or all of the studies. The lowest relevant NOAELs after oral administration were 1000, 1000, and 600 ppm, equal to 160, 57, and 18mg/kg bw per day, respectively, for the three species (Pavela, 2004). The hepatotoxicity in mice, rats, and dogs showed increased liver weight with frequent changes in serum transaminase activity. In mice, increased liver weights were associated with a higher incidence of hepatocellular hypertrophy. In the livers of rats and dogs, generally unremarkable histopathological changes were observed. At doses of 85mg/kg bw per day and above, a pyrethrum extract containing 20% pyrethrins induced an increase in microsomal enzymes in rats. Furthermore, anaemia was observed in rats and dogs at doses of 3000 ppm and above. The kidney was another target, but only in rats. In the 13-week study, rats at doses greater than 1000 ppm had increased kidney weights associated with tubular degeneration and regeneration in the renal cortex (Pavela, 2004).

By changing the route of exposure to inhalation in rats, the NOAEL for systemic toxicity was 0.011 mg/L. The increases in liver weight were clearly related to exposure and were accompanied by changes in serum transaminase activity. Non-regenerative anaemia was also observed. The weights of the kidney and lung were increased in relation to body weight. The morphological abnormalities observed in the larynx, nasoturbinales, nasopharynx and lungs by light microscopy were considered to be localised responses indicative of a treatment-related effect (Pavela, 2004).

Dermal administration of pyrethrins at doses up to 1000 mg/kg bw per day for 21 days caused no systemic toxicity in rabbits. In a two-year study of toxicity and carcinogenicity in rats and an 18-month study of carcinogenicity in mice, the NOAEL was 100 ppm in both species, equal to 14 and 4 mg/kg bw per day in mice and rats, respectively. The liver was the main target. A treatment-related effect on the incidence of lung tumours was seen in mice and increased incidence of benign tumours of the skin, liver, and thyroid were observed in rats. The Meeting of Toxicologists (IPCS-INTOX) concluded that the increased tumour incidences caused by pyrethrins were threshold phenomena of negligible relevance to the low doses to which humans are exposed. The Meeting also concluded that pyrethrins had no genotoxic or mutagenic potential, but a test for gene mutation in mammalian cells is still lacking.

Pyrethrins did not show developmental toxicity in rats or rabbits at the highest maternally toxic doses tested, which were 75 and 250 mg/kg bw per day, respectively. The only effects on the offspring, observed in a two-generation study of reproductive toxicity in rats, were reduced body weights at the parentally toxic doses of 1000 and 3000ppm, with a NOAEL of 100 ppm, equivalent to 10 mg/kg bw per day (Pavela, 2004).

The available data on humans did not show a causal relationship between exposure to modern pyrethrin-containing products and significant adverse health effects (Pavela, 2004).

An average daily intake (ADI) of 0–0.04 mg/kg bw was established for the tested blend of refined pyrethrum extract, which was based on the NOAEL of 100 ppm, equal to 4 mg/kg bw per day, observed in the long-term study of toxicity and carcinogenicity in rats and a safety factor of 100. This figure is identical to the ADI derived by the 1972 Meeting, which was based on a NOAEL of 200ppm, equivalent to 10 mg/kg bw per day in a long-term study in rats and a safety factor of 250 (Pavela, 2004).

### Shankpushpi Powder (Scientific Name: *Convolvulus pluricaulis*)

The Shankpushpi plant is known for its medicinal properties. It is used traditionally to treat nervous debility, insomnia, fatigue, and low energy level. The whole herb is used medicinally in the form of decoction with cumin and milk in fever, nervous debility and loss of memory. It is also used as a sovereign remedy in bowel complaints, especially dysentery. The ethanolic extract of the plant reduces total serum cholesterol, triglycerides, phospholipids and nonesterfied fatty acids. The herb appears to produce its action by modulation of brain neurochemistry. Further, the herb is non-toxic and its use does not produce any side effects. On the other hand, there is an invigorating effect in improvement of health and weight gain. Chemical studies of the whole plant have shown the presence of glycosides, coumarins, flavonoids and alkaloids. Shankha pushpine, (the alkaloid) has been identified as active chemical. B. sitosterol glycoside, hydroxy cinnamic acid, octacosanol tetracosane along with glucose, sucrose also have been isolated from the plant (Indian Medicinal Plants, 2006).

#### Toxicology

The LD_50_ of whole extract of Convolvulus pluricaulis is reported to be 1250 mg/kg b.w. in mice. Mice treated with the extract of Convolvulus pluricaulis at doses of more than 200 mg/kg b.w. showed sedative effects and reflected a moderate to marked decrease in locomotor activity which lasted nearly 12 hrs (Pawar *et al.*, 2001).

### Sabja powder (Ocimum basilicum)

*Ocimum basilicum* L. is an aromatic herb which is known as Sabja in India. It is used to treat illnesses such as respiratory and rheumatic problems, vomiting, and pain (Venâncio *et al.*, 2011). It is also used as an antiinflammatory agent, blocking both cyclooxygenase and lipoxygenase pathways of arachidonic acid metabolism (Singh 1999). Further it possesses the larvicidal and repellent potential against the dengue vector Aedes aegypti (Insecta: Diptera: Culicidae) (Murugan *et al.*, 2006)**.**

#### Acute toxicity

In Swiss mice the lethal dose (LD50) is calculated as 532 mg/kg b.w.. There are only a few studies regarding acute toxicity and very little is known about the histomorphological changes produced in various vital organs by toxic doses of Ocimum basilicum (Venâncio *et al.*, 2011).

#### Chronic toxicity

Information on the matter was not available in sources used to compile this review.

### Nirgudi Powder (*Vitex negundo*)

*Vitex negundo* L. (VN) has been investigated extensively for its antiinflammatory and analgesic (Singh, 1999; Murugan *et al.*, 2006) activities. In addition, another group of researchers noticed the inhibitory activity of the extract on prostaglandin biosynthesis and confirmed NSAID-like activity (Telang *et al.,* 1999). But the type of cyclooxygenase inhibition produced by the extract is yet to be explored (Telang *et al.*, 1999). In traditional medicine it is also used to treat respiratory disorders. The antitussive effect of the butanolic extract of *V. negundo* (Vn) on sulphur dioxide (SO_2_)-induced cough was examined in mice (Haq *et al.*, 2011).

The Vitex species contains various active chemicals (like lauric acid, palmitic acid, stearic acid, oleic acid and linoleic acid). Its fatty acid methyl ester (FAME) extracts showed larvicidal activity against early fourth-instar larvae of Culex quinquefasciatus with an LC50 value of V. negundo (18.64 ppm) (Kannathasan *et al.*, 2008). The leaf hexane extract of V. negundo has the potential to be used as an ideal eco-friendly approach for the control of the A. subpictus and C. Tritaeniorhynchus by its mosquito larvicidal activity (Kamaraj *et al.*, 2009; Kamaraj *et al.*, 2010).

#### Acute toxicity

There are only a few studies regarding acute toxicity and very little is known about the histomorphological changes produced in various vital organs by toxic doses of *Vitex negundo*.

The oral LD_50_ dose of *Vitex negundo* leaf extract is 7.58g/kg, b.w. of rats.

#### Chronic toxicity

Dose-dependent histomorphological changes were observed in specimens of the heart, liver and lung. Toxic effect on the myocardium was noticed both with lower and higher doses. At microscopic examination, specimens of the heart appeared grossly thickened and hyperaemic and also showed vascular dilatation and haemorrhage significantly *(p<0.05)* at 2.5 and 5 g/kg, b.w. doses and *(p<0.01)* at 7.5 and 10 g/kg b.w. doses. With the dose 10g/kg b.w. also changes in the lung were observed (Tandon *et al.*, 2004). These findings are in agreement with those of Ravishankar *et al.* (1985).

### Karanj powder (Scientific Name: *Pongamia pinata*)

*Pongamia pinnata* (Linn.) locally known as Karanja, is a small evergreen tree, which is widely distributed in Bangladesh, India, China the Philippines and Australia. All parts of the plant have been used as a crude drug for the treatment of tumours, piles, skin diseases, itches, abscesses, painful rheumatic joints, wounds, ulcers, diarrhoea, etc. (Meera *et al.*, 2003; Shoba & Thomas, 2001). Besides, it is well known for its application as animal fodder, green manure, timber and fish poison. It has also been recognized to provided applications in agriculture and environmental management, with insecticidal and nematocidal activity (Brijesh *et al.*, 2006). Seed oil is used in scabies, leprosy, piles, ulcers, chronic fever. Limited data are available on the uses of Karanja (Marathi) or Karuaini (Hindi) plant as a pesticide. A study showed the oil of the Pongamia pinata to be active against the spider mite (Kumar *et al.*, 2003).

The results of the study conducted by Srinivasan *et al.* (2003) showed that the 70% ethanolic extract of Pongamia pinnata leaves possessed marked antinociceptive as well as antipyretic activities and were thus scientifically validated in the treatment of pain and pyretic disorders. The 70% ethanolic extract of Pongamia pinnata leaves did not show any sign of toxicity and mortality up to a dose level of 10.125 g/kg, p.o. in mice (Srinivasan *et al.*, 2001).

Extracts of the stem and roots of Pongamia pinnata showed moderate larvicidal effects after 24 h exposure at 1,000 ppm. (Rahuman *et al.*, 2009).

#### Acute toxicity

The acute oral toxicity studies of the extracts were carried out by administration of stepwise doses of all four extracts of *P. pinnata* from 50 mg/kg b. wt. up to a dose of 5000mg/kg b.w. There were no considerable signs of toxicity in the animals tested. One-tenth of the upper limit dose was selected as the level for examination of antidiabetic activity (Singh *et al.*, 1997).

#### Chronic toxicity study

None of the acute toxicity studies of extracts of *P. pinnata* leaves showed any significant toxicity when observed during the first 4 hrs. Daily observations for 14 days showed no mortality. The drug was found to be safe at the tested dose level of 5000 mg/kg b. w. One-tenth of this dose level was considered as effective dose (Mukesh *et al.*, 2010).

### Sal (Sakhu) (Scientific Name: *Shorea robusta*)

*Shorea robusta* resin has a stronger and broader spectrum of antimicrobial activity against a number of pathogenic microorganisms and the extracts may be used to discover bioactive natural products that may serve as basic source for the development of new drugs for the therapy of skin allergies, diarrhoea, dysentery and astringency (Murthy *et al.*, 2011) . The results obtained also provide support to uses of the plants in traditional medicine. However, before their administration, toxicological analysis of the active compounds is necessary in order to assess their tolerance in the human body.

#### Toxicity

Information on acute/chronic toxic effects due to Sakhu (Shorea robusta**)** are not available on Google, PUBMED, IPSC-INTOX, Scopus, etc.

### Karamkas (Scientific Name: *Butea frondosa*)

In India, this herb is known as kamarkas, which means fortification of back muscles; it acts as a tonic to pelvic and back muscles during menstruation, pregnancy and after delivery. The gum of this herb contains mainly tannins and gallic acid and for this reason it is used as a mild astringent useful in phthisis and haemorrhage of the stomach and also as an antihelmintic. The plant is used as refreshment and sterile for promoting urination and is useful in threadworm infections (Gurpreet *et al.*, 2011).

In folk medicine, *Butea frondosa* is being used as an antidiarrhetic. Although the stem bark extract of *Butea frondosa* was found to possess antidiarrhoeal activity, it is important to suggest that every part of the plant is unique in function and is endowed with a different proportion of active constituents and therefore might possess different therapeutic qualities. The rationality of using the leaves is substantiated, as the leaf extract has been evaluated for its antiinflammatory action. It has also been traditionally used as an astringent, in colic, against worms and in piles (Nandkarni AK, Chopra RN 1976) . *Butea frondosa* is bestowed with flavanoids, glucosides and lectins (Soman *et al.*, 2004). It was are reported to possess nootropic and antistress properties (Mengi& Deshpande, 1995).

#### Toxicity

Information on acute/chronic toxic effects of *Butea frondosa* is lacking.

### Soapstone powder (Scientific Name: *Magnesium Silicate*)

Trade Names: Baby Powder, Talc, Talcum, Magnesium Silicate Hydrate, Magnesium Silicate Hydroxide, Steatite, Slab, and Tile**.** Only limited information is available on acute/ chronic toxic effects of soapstone powder.

#### Acute toxicity

**Dermal:** Direct contact may cause dryness, irritation by mechanical abrasion, or skin allergies. Skin absorption usually is not a significant route of exposure.

**Eyes:** Direct contact with dust while cutting or removing debris may cause eye irritation by mechanical abrasion, with discomfort or pain, local redness, and swelling of the conjunctiva.

**Inhalation:** If inhaled in the form of dust, it may cause nose, throat, and respiratory tract irritation by mechanical abrasion or corrosive action. Exposures in excess may cause coughing, sneezing, chest pain, shortness of breath, and inflammation of mucous membrane, due to mechanical irritation. One also may experience a flu-like fever.

**Ingestion:** If a small amount (a tablespoonful) is swallowed during normal handling, it is not likely to cause injury. Ingestion of large amounts may cause gastrointestinal irritation and/or blockage. Use of soapstone for construction purposes should not cause acute toxic effects. However, inhaling dust may aggravate existing respiratory system disease(s) and/or dysfunctions.

#### Chronic toxicity

Soapstone usually contains less than 2% silica. Exposure to silica containing dust poses a potential health hazard. Repeated overexposure to very high levels of respirable crystalline silica (quartz, cristobalite, tridymite) for periods of six months or more have caused acute silicosis, and repeated exposure to dust may result in talc pneumoconiosis. Not all individuals with silicosis will exhibit symptoms (signs) of the disease. But symptoms can appear at any time, even years after exposure had ceased. Symptoms include (but are not limited to): shortness of breath, diminished work capacity, cough, fever, right heart enlargement and/or failure, weight loss, and chest pain.

Excessive inhalation of dust may result in respiratory disease, including silicosis, pneumoconiosis, and pulmonary fibrosis (scarring of the lungs). Persons with silicosis have an increased risk of pulmonary tuberculosis infection. Smoking may increase the risk of developing lung disorders, including emphysema and lung cancer. Respirable dust containing newly broken silica particles has been shown to be more hazardous to animals in laboratory tests than respirable dust containing older silica particles of similar size (Bergman, 2006).

## Conclusions

Repellents do not all share a single mode of action and surprisingly little is known about how repellents act on their target insects. Moreover, different species of Mosquito may react differently to the same repellent. The “Mosquito Out” powder includes different plant powders which act on different vital systems of the insects. Thus Neem powder (acts on reproductive system) has been shown to inhibit of larval, pupal and adult moults and reproduction and fitness of both plant feeding and aquatic larvae of mosquitoes. It was not found toxic up to the dose of 1200 mg/kg b.w. It was given orally in order to evaluate the No-Observed-Effect-Level (NOEL) of the active principle (Azadirachtin) of Neem to calculate its safety margin. The composition of the powder contains only a total of 2000 mg of Neem powder, which can produce acute toxicity if the whole powder is administered orally to an animal whose weight is 1 kg. According to the WHO grading on the basis of LD50, such poisonous substances come under Non-Toxic Chemicals.

After analysis of available data and information on the ingredients of the product in relation to medicinal uses, acute and chronic toxicity of the selected medicinal plants, it can be concluded that no toxic effects of extracts of these selected plants were reported in mammals. This may be due to the small amounts, species specificity, and affinity characteristics of the agents/ active chemicals. The findings lead to the conclusion that if the product which contains the powder of the above said plants is applied with care and safety as a mosquito repellent/killer its use should not be discouraged. The conclusions drawn in this article are totally based on the information and data generated by other researchers. Therefore the author of this article recommends to conduct studies to evaluate the potential toxic hazards of the product.
